# A dual attention and cross layer fusion network with a hybrid CNN and transformer architecture for medical image segmentation

**DOI:** 10.1038/s41598-025-19563-w

**Published:** 2025-10-13

**Authors:** Jiahong Chen, Zhengyou Liang, Xiangyan Lu

**Affiliations:** 1 School of Computer, Electronics and Information, Guangxi University, Nanning, 530004 China; 2 Guangxi Key Laboratory of Multimedia Communications and Network Technology, Nanning, 530004 China; 3 Parallel and Distributed Laboratory, Guangxi University, Nanning, 530004 China

**Keywords:** Medical image segmentation, Deep learning, Feature fusion, Attention mechanism, Computational biology and bioinformatics, Engineering, Mathematics and computing

## Abstract

Medical image segmentation is a crucial technology for disease diagnosis and treatment planning. However, current approaches face challenges in capturing global semantic dependencies and integrating cross-layer features. While Convolutional Neural Networks (CNNs) excel at extracting local features, they struggle with long-range dependencies; Transformers effectively model global context but may compromise spatial details. To address these limitations, this paper proposes a novel hybrid CNN–Transformer architecture, Dual Attention and Cross-layer Fusion Network (DCF-Net). Based on an encoder–decoder framework, DCF-Net introduces two key modules: the Channel-Adaptive Sparse Attention (CASA) module and the Synergistic Skip-connection and Cross-layer Fusion (SSCF) module. Specifically, CASA enhances semantic modeling by filtering critical features and focusing on anatomically important regions, while SSCF enables effective hierarchical feature fusion by bridging encoder–decoder representations. Extensive experiments on the Synapse, ACDC, and ISIC2017 datasets demonstrate that DCF-Net achieves state-of-the-art performance without pre-training. This work highlights the value of cross-layer fusion and attention mechanism, providing a robust and generalizable solution for medical image segmentation tasks.

## Introduction

Medical image segmentation is a foundational component of computer-aided diagnosis (CAD), aiming to identify critical anatomical structures such as organs, tumors, and lesions through pixel-level classification^1^. As a core technique in clinical practice, it is essential for diagnosing diseases, planning surgeries, and monitoring treatment^2^. Owing to their capacity to learn complex imaging features, deep learning models have been widely applied to medical image segmentation, leading to significant improvements in segmentation accuracy^3^. In the field of semantic segmentation, global context modeling, local detail encoding, and long-range dependency modeling are crucial. Recent advancements in these areas^4,5^ have provided valuable guidance for medical image segmentation. Nevertheless, compared to semantic segmentation in natural images, medical image segmentation still faces inherent limitations. First, medical images are typically produced by devices such as MRI and CT scanners, which often result in single-channel images with less rich raw information compared to natural images. Second, the anatomical structures in medical images generally present characteristics such as weak boundaries^6^, low contrast^7^, and irregular shapes^8^, which make segmentation tasks more challenging. These difficulties have driven the research community to explore more powerful contextual modeling mechanisms and more effective cross-layer feature fusion strategies.

During the early development of medical image segmentation, the U-shaped convolutional neural network (CNN) architecture became the dominant framework for this task^9^, exemplified by U-Net^10^. To enhance the capabilities of CNNs and U-Net variants, a wide range of modifications have been proposed. These include diverse skip connection strategies^11,12^, innovative convolutional blocks^13,14^, attention mechanisms^15–17^, and multi-scale feature extraction techniques^18,19^. However, CNN-based methods are inherently limited by local receptive fields and struggle to model long-range dependencies, making it difficult to accurately segment large anatomical regions.

Transformers are effective in capturing global contextual information through self-attention, showing notable advantages in modeling long-range dependencies^20^. Building on the success of the Vision Transformer (ViT)^21^, recent research has centered around two main architectural paradigms: hybrid CNN–Transformer models and pure Transformer-based architectures. Hybrid models such as TransUNet^22^, CoTr^23^, and HiFormer^24^ combine the local modeling capabilities of CNNs with the long-range dependency strengths of Transformers, aiming to overcome their respective limitations. In contrast, approaches like MERIT^25^, MISSFormer^26^, and TransDeepLab^27^ employ fully Transformer-based architectures, constructing both encoders and decoders using Transformer blocks and relying exclusively on self-attention mechanisms. Compared to hybrid CNN–Transformer models, pure Transformer methods excel at modeling long-range dependencies but lack the intrinsic local inductive bias of CNNs, limiting their ability to capture fine-grained spatial details.

Despite recent progress, current medical image segmentation methods still face two major challenges. First, the attention mechanisms in decoder modules are often uniform, relying on a single type of attention mechanism. This lack of diversity limits the model’s ability to exploit the complementary strengths of different attention mechanisms. Second, feature fusion modules in most frameworks are often inefficient, limiting the ability to model complex cross-hierarchical dependencies between low-level details and high-level semantics. To this end, we propose a novel hybrid CNN–Transformer network architecture, namely the Dual-attention Cross-layer Fusion Network (DCF-Net). DCF-Net employs a CNN encoder for capturing local detail features and innovatively integrates a Channel-Adaptive Sparse Attention (CASA) module in the decoder. CASA enables synergistic modeling of global context and long-range dependencies. Additionally, we design a Synergistic Skip-connections and Cross-layer Fusion (SSCF) module to intelligently fuse high-resolution shallow features from the encoder with deep semantic information from the decoder, thereby enhancing representation learning for complex anatomical regions. In summary, our contributions are as follows:

- Efficient hybrid architecture design: We propose DCF-Net, a hybrid framework combining convolutional neural networks (CNNs) and Transformers. It achieves a balance between local detail preservation and global context modeling without relying on pre-training, and effectively segments complex anatomical structures with irregular boundaries or those characterized solely by edge information.
- Dual attention mechanism: The Channel-Adaptive Sparse Attention (CASA) module integrates Cross-Covariance Attention (XCA) and Top-k Sparse Attention (TKSA) in a cascaded manner. This module not only captures global interactions along the channel dimension to filter critical semantic features but also dynamically focuses on anatomically salient regions through sparsification, thereby addressing the limitations of existing single-dimensional attention mechanisms in terms of channel sensitivity and feature redundancy.
- Cross-layer feature fusion: The Synergistic Skip-Connections and Cross-layer Fusion (SSCF) module employs dual residual enhancement combined with a lightweight compression strategy. This design refines shallow encoder features and deep decoder semantics, thereby improving the representation of fine anatomical structures.
- Extensive empirical validation: We conduct comprehensive experiments on three public medical image segmentation datasets, namely Synapse^28^, ACDC^29^, and ISIC2017^30^, to evaluate the effectiveness and generalizability of our method across diverse anatomical structures and imaging modalities. These experiments offer novel insights into the design and optimization of hybrid CNN–Transformer architectures.

## Related work

### Medical image segmentation

The field of medical image segmentation has undergone substantial evolution in recent years, transitioning from CNN-based methods to Transformer-based architectures. Motivated by the success of U-Net^10^, encoder–decoder architectures with skip connections have become a dominant CNN-based framework in medical image segmentation. However, due to the inherent limitation of the localized receptive field in convolutional operations, these models still struggle to effectively capture long-range dependencies. The introduction of the Vision Transformer (ViT)^21^ has spurred extensive research on applying Transformer architectures to medical image segmentation. TransUNet^22^ was the first to introduce a hybrid framework that integrates convolutional operations with self-attention, enabling joint modeling of local and global features. Some studies aim to capture long-range spatial context through pure Transformer-based approaches, thereby partially expanding the receptive field of the network. Specifically, Swin-UNet^31^ utilizes the Swin Transformer^32^, removing convolution entirely while retaining strong global modeling capabilities.

Recent advances in hybrid architectures have demonstrated significant progress across diverse domains. TransXNet^33^ addressed bottlenecks in existing hybrids by proposing a lightweight Dual Dynamic Token Mixer (D-Mixer), dynamically integrating global-local features for superior image processing. ATC-Net^34^ proposed a dual-branch encoder (Transformer + CNN) with feature-enhanced fusion modules, effectively aggregating global context and local details for optical remote sensing salient object detection (SOD). IFENet^35^ advanced multi-modal SOD through a “Interaction-Fusion-Enhancement” pipeline, combining graph-based modality interaction, 3D gated fusion, and frequency-domain enhancement within a Transformer-CNN framework.

Collectively, hybrid architectures surpass pure Transformers by preserving CNNs’ intrinsic local inductive bias while effectively modeling long-range dependencies. Inspired by these advances, we propose a novel hybrid CNN–Transformer architecture. Unlike previous hybrid models, our approach further optimizes the attention mechanisms and skip-connection design, facilitating a more effective fusion of the respective strengths of CNNs and Transformers.

### Attention mechanism

In medical image segmentation, attention mechanisms have been extensively employed to enhance model performance, particularly when dealing with anatomically heterogeneous structures with low contrast^36^. These mechanisms primarily comprise channel attention and spatial attention. CNN-based methods typically construct attention modules using convolutional variants. EMCAD^19^ integrates grouped large-kernel gated attention to enhance multi-scale representation, while MFSE-Net^37^ employs a Large-kernel Grouped Deformable Attention (LGDA) module to capture adaptive spatial contexts through deformable convolutions. In contrast, Transformer-based methods refine self-attention mechanisms to improve feature representation. UCTransNet^38^ enhances feature representation by refining self-attention mechanisms through channel-wise cross-attention modules, which enable multi-scale fusion and semantic alignment. ParaTransCNN^39^ leverages channel attention modules to consolidate information extracted locally through the CNN branch and modeled globally via the transformer branch, enabling value-aware propagation from encoders to decoders. To alleviate the computational demands associated with self-attention, MISSFormer^26^ employs an Efficient Self-Attention mechanism to effectively enhancing global dependency modeling and local context encoding.

However, existing approaches primarily focus on single-dimensional representations, limiting their ability to adaptively identify key anatomical structures. Even dual-attention fusion models^40–42^, which incorporate both spatial and channel dimensions, often fail to fully capture channel-wise semantic information during decoding, resulting in limited channel sensitivity and feature redundancy. To address this challenge, we propose the CASA module to enhance inter-channel semantic sensitivity. CASA not only establishes semantic associations for anatomical structures in the global channel dimension but also implements a dynamic sparse strategy based on channel response intensity, allowing the model to adaptively focus on key regions while suppressing detail degradation.

### Skip-connections

Skip-connections serve as a fundamental component of encoder-decoder architectures in medical image segmentation, enabling multi-scale feature propagation across layers to mitigate spatial detail loss during downsampling^43^. Existing methods primarily focus on expanding the number of connections via nested dense skip-connections^11^ or full-scale skip pathways^12^, or preprocessing encoder feature maps^44^. However, simplistic fusion operations (e.g., addition/concatenation) commonly lead to redundant semantic aggregation and fail to dynamically model cross-layer dependencies. Although transformer-based approaches^38,45^ introduced cross-attention into skip-connections, their single-stage attention computation still inadequately leverages shallow-deep feature synergy, limiting complex anatomical structure perception.

To overcome this limitation, we introduce the SSCF module to facilitate cross-layer feature calibration and multi-scale aggregation, effectively bridging the semantic gap between CNNs and Transformers and enhances structural perception. Unlike Dynamic Multi-layer Channel Aggregator^46^, which dynamically aggregates features from multiple preceding layers, our SSCF focuses specifically on cross-layer fusion between encoder and decoder stages, enhancing the skip-connection design in U-shaped architectures to effectively integrate deep semantic information with shallow visual details.

## Methodology

This section systematically presents the DCF-Net architecture, focusing on its core innovative modules: CASA and SSCF. The overall architecture of DCF-Net is introduced in the first part. Next, the cascaded CASA architecture is detailed, which integrates Cross-covariance Attention (XCA) and Top-k Sparse Attention (TKSA) to achieve dual-attention synergy. Finally, the SSCF module, reconstructed from the Spatial and Channel Synergistic Attention (SCSA) module, is introduced and innovatively deployed on skip-connection paths for cross-layer heterogeneous feature fusion.

**Fig. 1:**
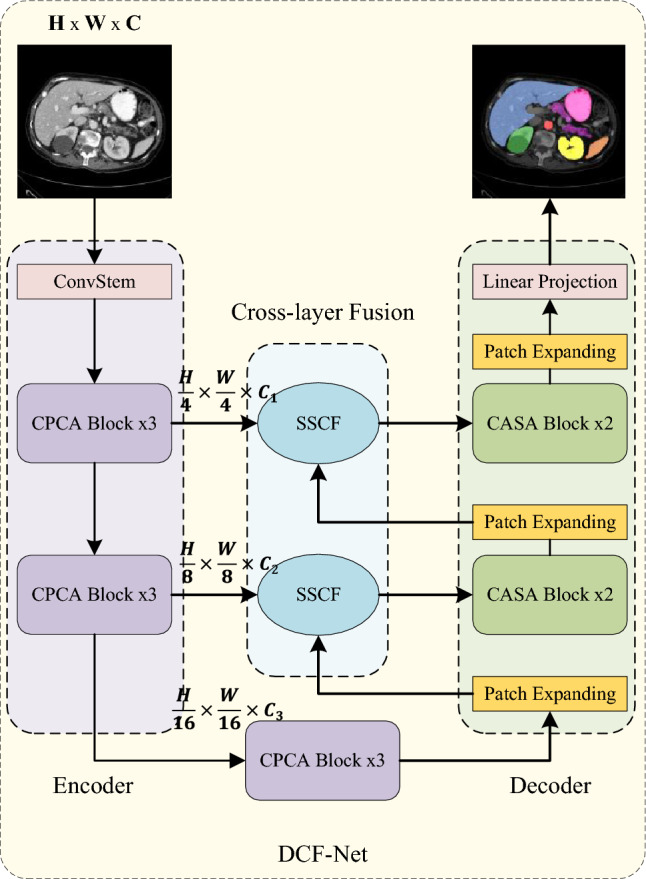
Overview of the proposed DCF-Net architecture.

### Overall architecture

The overall architecture of DCF-Net is depicted in Fig. 1. Specifically, The framework comprises consists of three core components: a CNN-based Encoder, a Dual-Attention Decoder, and a Cross-layer Fusion Module. Given an input image $x\in \mathbb {R}^{H\times W\times C}$, the encoder employs three stacked CPCA-Blocks^36^ per stage across three hierarchical levels, generating multi-scale feature maps with resolutions of $\frac{H}{4}\times \frac{W}{4}$, $\frac{H}{8}\times \frac{W}{8}$, $\frac{H}{16}\times \frac{W}{16}$, respectively. In the decoder, features from lower stages or the bottleneck are first upsampled via a patch expanding layer, which halves the number of channels and doubles the spatial resolution. The upsampled feature maps are then fused with the corresponding encoder feature maps at the same scale via the SSCF module to achieve heterogeneous cross-layer fusion. Following this, the integrated features are refined by two consecutive CASA Blocks to perform channel–spatial recalibration. Finally, the output from the topmost decoder stage is mapped to pixel-wise predictions through a linear projection layer, yielding segmentation masks at the original input resolution $H\times W$.

### Channel-adaptive sparse attention (CASA)

**Fig. 2:**
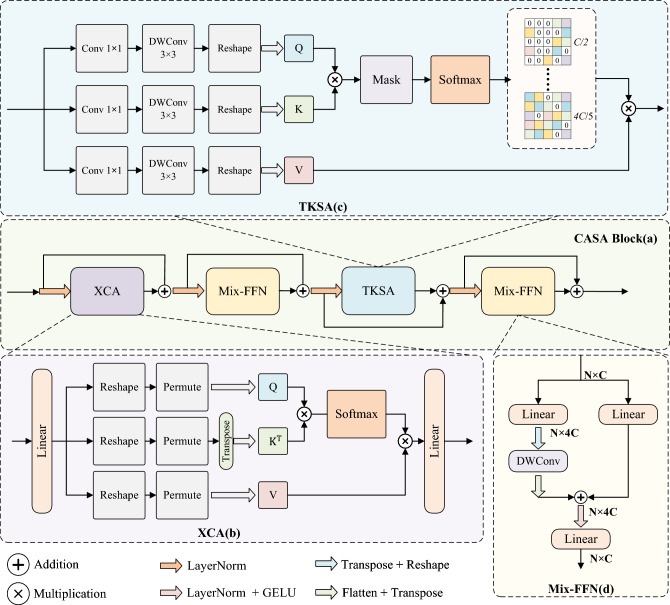
Structure of Channel-Adaptive Sparse Attention (CASA) Module. (**a**) Overview of the CASA Block; (**b**) Structure of the Cross-Covariance Attention (XCA) module; (**c**) Structure of the Top-k Sparse Attention (TKSA) module; (**d**) Structure of the Mix-FFN module.

Inspired by TKSA^47^ and XCA^48^, we propose a dual-attention module termed CASA, as the core feature extraction unit. Its structural design is illustrated in Fig. 2.

We observe that the feature maps produced by the CNN encoder encode rich semantic information along the channel dimension. However, repeated downsampling operations inevitably lead to channel expansion, which introduces semantic redundancy and noise. To enhance inter-channel dependencies and extract discriminative features, the CASA module initially feeds these semantically enriched feature maps into the XCA mechanism. This attention operation selectively filters channel-wise semantics, thereby suppressing non-essential responses and preserving critical information. Nevertheless, conventional attention computation requires pairwise similarity estimation between all query–key pairs, implying that even features intended to be suppressed are still incorporated into the attention process. This leads to computational redundancy and hampers the extraction of salient information. In the context of medical image segmentation, background regions frequently lack discriminative value for organ or lesion delineation. To address this, we further process features using TKSA, which introduces a sparsification mechanism that enables the model to concentrate on the most relevant segmentation regions.

For feature maps $\textbf{X}_{in}$ obtained from the encoder or lower decoder layers, the CASA decoding process can be formulated as follows:

1 $$\begin{aligned} \textbf{X}_c = \textbf{X}_{\textrm{in}} + \text {XCA}\left( \text {LN}\left( \textbf{X}_{\textrm{in}}\right) \right) , \end{aligned}$$

2 $$\begin{aligned} \textbf{X}_c' = \textbf{X}_c + \text {MixFFN}\left( \text {LN}\left( \textbf{X}_c\right) \right) , \end{aligned}$$

3 $$\begin{aligned} \textbf{X}_s = \textbf{X}_c' + \text {TKSA}\left( \text {LN}\left( \textbf{X}_c'\right) \right) , \end{aligned}$$

4 $$\begin{aligned} \textbf{X}_{\text {out}} = \textbf{X}_s + \text {MixFFN}\left( \text {LN}\left( \textbf{X}_s\right) \right) . \end{aligned}$$

LN denotes layer normalization; $\textbf{X}_c$ and $\textbf{X}_s$ represent the residual outputs after XCA and TKSA, respectively, while $\textbf{X}_c^\prime$ and $\textbf{X}_{\text {out}}$ denote the corresponding outputs after being further refined by the Mix-FFN network.Unlike the standard multilayer perceptron (MLP) used in traditional self-attention mechanisms, we adopt the feedforward network design from SegFormer^49^, which integrates skip connections and lightweight convolutions to better aggregate local features in the decoder. The corresponding computational process is formulated as follows:

5 $$\begin{aligned} \text {MixFFN}(x) = \text {FC}_2\left( \sigma \left( \text {LN}\left( \text {DWConv}\left( \text {FC}_1(x)\right) \oplus \text {FC}_1(x)\right) \right) \right) \end{aligned}$$

where $\text {FC}_1$ and $\text {FC}_2$ denote linear fully connected layers, $\text {DWConv}$ represents depthwise separable convolution, $\sigma$ refers to the GELU activation function, and $\oplus$ indicates element-wise addition.

Overall, the CASA module, which integrates XCA and TKSA, establishes semantic associations for anatomical structures at the global channel level and applies a dynamic sparse strategy based on channel response intensity. It dynamically calibrates feature responses, enhancing semantic relevance while suppressing redundant features.

#### Cross-covariance attention (XCA)

XCA is a channel attention mechanism based on transposed attention, with its core innovation lying in establishing global interactions along the channel dimension via cross-covariance matrices, rather than along the traditional spatial dimension. Given input feature matrices $Q \in \mathbb {R}^{N \times d}$, $K \in \mathbb {R}^{N \times d}$, and $V \in \mathbb {R}^{N \times d}$, they are first transposed. The cross-covariance matrix is then computed as:

6 $$\begin{aligned} \text {XCA}(Q, K, V) = V \cdot \text {Softmax}\left( \frac{K^\top Q}{\tau }\right) \end{aligned}$$

To enhance training stability, *Q* and *K* are subjected to $\ell _2$ normalization, ensuring that the elements of the resulting cross-covariance matrix lie within the range $(-1, 1)$. To mitigate potential limitations on feature expression imposed by $\ell _2$ normalization, a learnable temperature parameter $\tau$ is introduced to facilitate adaptive feature scaling.

#### Top-k sparse attention (TKSA)

TKSA first computes the similarity matrix $S = (Q \cdot K^\top ) \odot \tau$ from the given query matrix *Q* and key matrix *K*, where $\odot$ denotes element-wise scaling by a temperature factor $\tau$. It then generates a sparse attention mask via the Top-*k* selection operator:

7 $$\begin{aligned} \text {MASK}_k(S)_{ij} = {\left\{ \begin{array}{ll} S_{ij}, & \text {if } S_{ij} \ge S_{i,k} \\ -\infty , & \text { otherwise } \end{array}\right. } \end{aligned}$$

where $S_{i,[k]}$ denotes the *k*-th largest value in the *i*-th row of *S*. The parameter *k* is dynamically adjusted within the interval $[C/2,\ 4C/5]$ to adaptively control sparsity for segmenting organs of varying sizes. The sparsified matrix $\text {MASK}_k$ is then normalized using the Softmax function and aggregated with the value matrix *V*:

8 $$\begin{aligned} \text {Att}_k = \text {Softmax}\left( \text {MASK}_k(S)\right) \cdot V \end{aligned}$$

To further enhance multi-scale feature representation capability, TKSA employs a multi-head attention mechanism that performs parallel computation of local and global features. Attention outputs at four distinct sparsity levels ($k = C/2,\ 2C/3,\ 3C/4,\ 4C/5$) are integrated using learnable weights $\{\alpha _1, \alpha _2, \alpha _3, \alpha _4\}$:

9 $$\begin{aligned} \text {Output} = \sum _{i=1}^{4} \alpha _i \cdot \text {Att}_{k_i} \end{aligned}$$

### Synergistic skip-connections and cross-layer fusion (SSCF)

SCSA ^50^ achieves synergistic enhancement of multi-semantic features by jointly modeling spatial and channel attentions through two sequential stages: Shareable Multi-semantic Spatial Attention (SMSA), which extracts multi-scale features from critical spatial regions to guide subsequent channel attention, and Progressive Channel Self-attention (PCSA), which mitigates semantic inconsistencies via adaptive feature interaction. This two-stage design establishes a bidirectional optimization mechanism.

While integrating SCSA as an independent module effectively captures spatial–channel relationships within single-layer features, its application remains confined to this context, limiting its potential for hierarchical semantic fusion. To address this, we redefine the role of SCSA by embedding it within the skip-connections of U-shaped architectures. This reconfiguration transforms SCSA from a single-level feature enhancement unit into a central component for cross-layer feature fusion, thereby enabling semantic interaction across hierarchical levels. Based on this reformulation, we design the SSCF module.

**Fig. 3:**
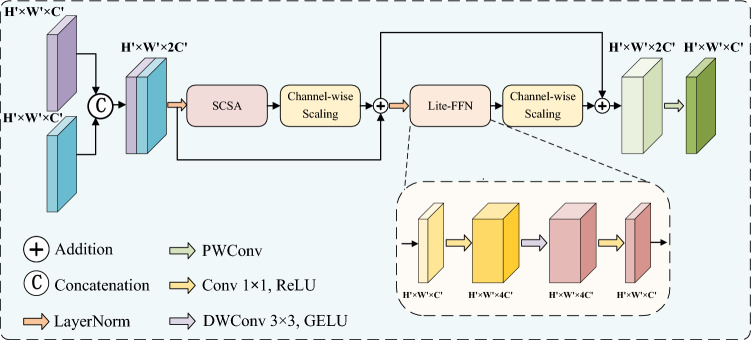
Structure of Synergistic Skip-connections and Cross-layer Fusion (SSCF) Module.

The proposed SSCF module facilitates multi-level semantic alignment across encoder–decoder skip-connections by integrating the SCSA mechanism with a lightweight architectural redesign. As shown in Fig. 3, SSCF processes encoder shallow features $x_1$ and decoder deep features $x_2$ through three consecutive stages: cross-layer feature concatenation, dual residual-enhanced refinement, and lightweight channel compression.

Initially, the shallow encoder features $x_1 \in \mathbb {R}^{B \times C \times H \times W}$ and the deep decoder features $x_2 \in \mathbb {R}^{B \times C \times H \times W}$ are concatenated along the channel dimension to construct a multi-semantic fusion feature map:

10 $$\begin{aligned} x_{\text {cat}} = \text {Concat}(x_1, x_2) \in \mathbb {R}^{B \times 2C \times H \times W}\text {.} \end{aligned}$$

The concatenated features undergo refinement via a dual residual enhancement mechanism implemented through the SCSA-Block. In the first residual branch, the SCSA module dynamically calibrates cross-layer dependencies by generating attention maps using multi-scale depthwise convolutions and channel shuffle operations, thereby enhancing discriminative regions while suppressing noise. The second residual branch incorporates a Lightweight Feed-Forward Network (Lite-FFN) to enhance nonlinear representation capacity. The computation within the SCSA-Block is formulated as:

11 $$\begin{aligned} x_s = x_{\text {cat}} + \gamma _1 \cdot \text {SCSA} \left( \text {LN}(x_{\text {cat}}) \right) , \end{aligned}$$

12 $$\begin{aligned} x_f = x_s + \gamma _2 \cdot \text {LiteFFN} \left( \text {LN}(x_s) \right) , \end{aligned}$$

Here, $\gamma _1$ and $\gamma _2$ are learnable channel-wise scaling parameters, initialized to small values, which control the residual strength of each branch. Layer Normalization is employed to ensure stable gradient propagation. To better leverage the rich shallow spatial features from the CNN encoder and effectively guide the deep semantic representations in the decoder, we adopt the lightweight convolutional operator Lite-FFN as the core unit of the feed-forward network within the SCSA-Block. Lite-FFN is formally defined as:

13 $$\begin{aligned} \text {LiteFFN}(x) = \text {Conv}_{1 \times 1}\left( \text {GELU} \left( \text {DWConv} \left( \text {Conv}_{1 \times 1}(x) \right) \right) \right) . \end{aligned}$$

Finally, a pointwise convolution is applied to lightly reduce the channel dimensionality, aligning the feature map with the target output shape while preserving critical information and reducing computational redundancy:

14 $$\begin{aligned} x_{\text {out}} = \text {PWConv}(x_f) \in \mathbb {R}^{B \times C \times H \times W}\text {.} \end{aligned}$$

In summary, the SSCF module bridges the semantic gap between the encoder and decoder through multi-stage residual interaction and lightweight channel compression, effectively integrating low-level spatial textures with high-level semantic context.

### Loss function

In this work, we employ a hybrid loss function consisting of Dice Loss and Cross-Entropy Loss, defined as:

15 $$\begin{aligned} \text {Loss} = \lambda \, L_{\text {CE}} + (1 - \lambda ) \, L_{\text {Dice}} \end{aligned}$$

where $\lambda$ is a weight parameter chosen based on the specific task. The formulas for Cross-Entropy Loss ($L_{\text {CE}}$) and Dice Loss ($L_{\text {Dice}}$) are as follows Eqs. (16) and (17):

16 $$\begin{aligned} L_{\text {CE}} = -\frac{1}{N} \sum _{i=1}^{N} \sum _{c=1}^{C} L_{i,c} \log (P_{i,c}) \end{aligned}$$

Here, $N$ denotes the total number of pixels, and $C$ represents the number of semantic classes. $L_{i,c}$ is the one-hot encoded ground truth indicating whether the $i$-th pixel belongs to class $c$, and $P_{i,c}$ is the predicted probability (after softmax normalization) that the $i$-th pixel belongs to class $c$.

17 $$\begin{aligned} L_{\text {Dice}} = 1 - \frac{1}{C} \sum _{c=1}^{C} \frac{2 \sum _i P_{i,c} L_{i,c}}{\sum _i P_{i,c} + \sum _i L_{i,c}} \end{aligned}$$

Here, $C$ denotes the number of classes. $P_{i,c}$ and $L_{i,c}$ represent the predicted probability and the one-hot encoded ground truth for class $c$ at pixel $i$, respectively. The Dice loss is computed independently for each class and then averaged across all classes to obtain the final loss, effectively capturing the class-wise spatial overlap between the predictions and the ground truth.

## Experiments and results

### Datasets and preprocessing

We evaluate the performance of DCF-Net on three publicly available medical image segmentation datasets. For consistency and fair comparison across datasets, the preprocessing and data augmentation strategies for all datasets were based on prior works^9,22^. Input images from all datasets are uniformly resized to a resolution of $224 \times 224$.

Synapse Multi-Organ Segmentation (Synapse)^28^: This abdominal CT dataset consists of 30 clinical scans, with a total of 3,779 axial slices and pixel-level annotations for eight vital anatomical structures: spleen, right kidney, left kidney, gallbladder, liver, stomach, aorta, and pancreas. We used 18 cases (2,211 slices) for training and 12 cases (1,568 slices) for testing.

Automated Cardiac Diagnosis Challenge (ACDC)^29^: This cardiac MRI dataset includes short-axis cine sequences from 100 patients, covering both end-diastolic and end-systolic phases. Each case is annotated with precise contours of the right ventricle (RV), left ventricle (LV), and myocardium (MYO), resulting in a total of 1,902 slices. In line with standard practice, 70 cases (1,290 slices) are used for training, 10 cases (196 slices) for validation, and 20 cases (416 slices) for testing.

ISIC 2017 Skin Lesion Segmentation^30^: Released by the International Skin Imaging Collaboration (ISIC), this dermoscopic image dataset contains 2,000 high-resolution samples with expert-provided pixel-level segmentation masks, including common lesion types such as melanoma and benign nevi. The dataset is split into 1,400 training samples, 200 validation samples, and 400 testing samples.

### Implementing details

All experiments are conducted on an NVIDIA RTX 4080 GPU using the PyTorch 1.11.0 framework. To ensure fairness and consistency in our experimental setup, the optimization strategies for the datasets were configured according to previous works^9,22^. The batch size and learning rates were adjusted based on the GPU used, while other configurations such as optimizers remain consistent. For the Synapse and ACDC segmentation tasks, we adopt Stochastic Gradient Descent with Momentum (SGD) as the optimizer. The initial learning rate is set to $3.4 \times 10^{-3}$, with a momentum coefficient of 0.9 and a weight decay of $1 \times 10^{-4}$. The model is trained for 400 epochs with a batch size of 16, using $\lambda = 0.4$ in the loss function defined in Eq. (15). In contrast, for the ISIC 2017 segmentation task, we employ the Adam optimizer, with an initial learning rate of $1 \times 10^{-4}$. The model is trained for 100 epochs with a batch size of 4, using $\lambda = 0.5$ in the same hybrid loss function defined in (15).

### Evaluation indicators

For the Synapse and ACDC datasets, we employ two primary evaluation metrics: the Dice Similarity Coefficient (DSC) and the Hausdorff Distance (HD).

The DSC quantifies the spatial overlap between the predicted segmentation *P* and the ground truth label *L*, and is defined as:

18 $$\begin{aligned} \text {DSC} = \frac{2 \times |P \cap L|}{|P| + |L|} \end{aligned}$$

Here, $|P \cap L|$ denotes the number of overlapping pixels between the prediction and the ground truth. Higher DSC values indicate greater segmentation accuracy.

The HD measures the maximum distance between the boundaries of the prediction *P* and the ground truth *L*, and is defined as:

19 $$\begin{aligned} \text {HD} = \max \left\{ \sup _{a \in \partial P} \inf _{b \in \partial L} \Vert a - b \Vert ,\ \sup _{b \in \partial L} \inf _{a \in \partial P} \Vert b - a \Vert \right\} \end{aligned}$$

where *a* and *b* are points on the boundaries $\partial P$ and $\partial L$, respectively. Lower HD values indicate superior boundary consistency.

In addition to the Hausdorff Distance (HD), we also report HD95, which measures the 95th percentile of the Hausdorff distance between the predicted and ground truth boundaries. This metric focuses on the distance within which 95% of the boundary points fall, providing a more robust evaluation by reducing the influence of extreme outliers or noisy points.

For the ISIC 2017 dataset, we follow the evaluation protocol proposed by Azad et al.^9^ and incorporate five additional metrics commonly used in this context: Accuracy (AC), Precision (PR), Sensitivity (SE), Specificity (SP), and Intersection over Union (IoU). These are defined as follows:

20 $$\begin{aligned} \text {AC}&= \frac{TP + TN}{TP + FP + FN + TN} \end{aligned}$$

21 $$\begin{aligned} \text {PR}&= \frac{TP}{TP + FP} \end{aligned}$$

22 $$\begin{aligned} \text {SE}&= \frac{TP}{TP + FN} \end{aligned}$$

23 $$\begin{aligned} \text {SP}&= \frac{TN}{TN + FP} \end{aligned}$$

24 $$\begin{aligned} \text {IoU}&= \frac{TP}{TP + FP + FN} \end{aligned}$$

Here, TP (true positive), FP (false positive), FN (false negative), and TN (true negative) denote: correctly segmented target pixels, background pixels incorrectly classified as target, target pixels incorrectly classified as background, and correctly identified background pixels, respectively.

**Table 1:** Quantitative comparison between the proposed DCF-Net and state-of-the-art methods on the Synapse multi-organ segmentation dataset. The evaluation metrics are HD95 (mm) and DSC in (%).The number of model parameters is reported in millions (M), and computational efficiency is evaluated using FLOPs (in GFLOPs). $\uparrow$ ($\downarrow$) indicates higher (lower) values are better. “–” denotes unavailable results not reported in the corresponding source. Dice Similarity Coefficient (DSC) scores are reported for eight abdominal organs: aorta (Aor), gallbladder (Gal), left kidney (LKid), right kidney (RKid), liver (Liv), pancreas (Pan), spleen (Spl), and stomach (Sto). The best results for each organ are highlighted in bold, and the second-best results are underlined.

			Organ								Average	
Methods	Params	Flops	Aor	Gal	LKid	RKid	Liv	Pan	Spl	Sto	DSC $\uparrow$	HD95 $\downarrow$
TransUnet^22^	96.07M	88.91G	87.23	63.13	81.87	77.02	94.08	55.86	85.08	75.62	77.48	31.69
CTC-Net^44^	–	–	86.46	63.53	83.71	80.79	93.78	59.73	86.87	72.39	78.41	–
Swin-Unet^31^	27.17M	6.2G	85.47	66.53	83.28	79.61	94.29	56.58	90.66	76.60	79.13	21.55
TransDeepLab^27^	21.14M	15.68G	86.04	69.16	84.08	79.88	93.53	61.19	89.00	78.40	80.16	21.25
SelfReg-UNet^51^	–	–	86.07	69.65	85.12	82.58	94.18	61.08	87.42	78.22	80.54	–
HiFormer-L^24^	25.51M	17.76G	87.03	68.61	84.23	78.37	94.07	60.77	90.44	82.03	80.69	19.14
PVT-CASCADE^52^	34.12M	7.62G	83.01	70.59	82.23	80.37	94.08	64.43	90.10	83.69	81.06	20.23
VM-UNet^53^	–	–	86.40	69.41	86.16	82.76	94.17	58.80	89.51	81.40	81.08	19.21
MISSFormer^26^	42.46M	9.89G	86.99	68.65	85.21	82.00	94.41	65.67	91.92	80.81	81.96	18.20
DAEFormer^41^	48.07M	27.89G	87.84	71.65	87.66	82.39	95.08	63.93	91.82	80.77	82.63	16.39
PVT-GCASCADE^54^	24.86M	–	86.50	71.71	87.07	83.77	95.31	66.72	90.84	83.58	83.28	15.83
PAG-TransYnet^40^	–	–	**89.67**	68.89	86.74	**84.88**	**95.87**	68.75	92.01	80.66	83.43	15.82
ParaTransCNN^39^	45.70M	50.39G	88.12	68.97	**87.99**	83.84	95.01	69.79	**92.71**	84.43	83.86	15.86
**DCF-Net (ours)**	42.02M	18.47G	87.97	**72.67**	87.04	84.51	95.25	**70.37**	92.35	**85.22**	**84.44**	**14.02**

### Quantitative results

Tables 1–3 summarize the quantitative performance of DCF-Net on the Synapse, ACDC, and ISIC 2017 datasets, demonstrating that it achieves comparable or superior results to existing state-of-the-art methods. Specifically, on the Synapse dataset (see Table 1), we compared DCF-Net with several hybrid models, including TransUnet, CTC-Net, HiFormer, PVT-CASCADE, MISSFormer, PAG-TransYNet and ParaTransCNN. DCF-Net achieved the highest average Dice Similarity Coefficient (DSC) of 84.44%, outperforming all competing hybrid models. This result confirms the model’s effectiveness in cross-level feature fusion and its robustness in addressing the challenge of heterogeneous feature integration. Compared with DAEFormer, which also employs a dual-attention mechanism, DCF-Net improves the mean DSC by 1.81%, highlighting the superior feature-filtering capability of the proposed CASA module. Notably, DCF-Net achieves the best performance on typically challenging organs that are prone to low segmentation accuracy, including the gallbladder (Gal), pancreas (Pan), and stomach (Sto). This further demonstrates the CASA module’s ability to suppress irrelevant semantic noise, enhance anatomical saliency, and improve boundary localization in low-contrast or irregular structures. In addition, the model attains the lowest Hausdorff Distance (HD95), which is primarily attributed to the multi-stage feature interaction design of the proposed SSCF module. By deeply integrating high-resolution encoder textures with decoder-level semantic representations, SSCF effectively addresses heterogeneous feature alignment, resulting in segmentation contours that more closely conform to true anatomical boundaries.

**Table 2:** Quantitative comparison between the proposed DCF-Net and state-of-the-art methods on the ACDC cardiac segmentation dataset. The evaluation metrics are DSC in (%). $\uparrow$ indicates that higher values are better. Dice Similarity Coefficient (DSC) scores are reported for three cardiac structures: right ventricle (RV), myocardium (Myo), and left ventricle (LV). The best result for each structure is highlighted in bold.

Methods	RV	Myo	LV	Average DSC $\uparrow$
R50-VIT-CUP^22^	86.07	81.88	94.75	87.57
Unter^55^	85.29	86.52	94.02	88.61
TransUNet^22^	88.86	84.53	95.73	89.71
Swin-UNet^31^	88.55	85.62	95.83	90.00
LeViT-UNet-384^56^	89.55	87.64	93.76	90.32
MixedUNet^57^	86.64	89.04	95.62	90.43
SegFormer3D^58^	88.50	88.86	95.53	90.96
HiFormer-L^24^	89.58	88.28	95.47	91.11
MISSFormer^26^	89.85	88.38	95.34	91.19
DAEFormer^41^	89.92	88.84	95.21	91.33
ParaTransCNN^39^	89.19	89.07	95.80	91.35
SelfReg-UNet^51^	89.49	89.27	95.70	91.49
**DCF-Net (ours)**	**90.18**	**89.51**	**95.97**	**91.87**

On the ACDC dataset as reported in Table 2, DCF-Net achieves a mean DSC of 91.87%, surpassing all baseline methods across the segmentation of the right ventricle (RV), myocardium (Myo), and left ventricle (LV), with particularly strong performance in ventricular structures.

**Table 3:** Comparative analysis of the proposed DCF-Net against state-of-the-art methods on the ISIC 2017 dataset. The evaluation metrics are DSC in (%). The best result in each column is highlighted in bold.

Methods	AC	PR	SE	SP	Dice	IoU
U-Net^10^	0.9406	0.8762	0.5915	0.9785	0.7062	0.7409
Att-UNet^17^	0.9596	0.9012	0.8685	0.9794	0.8846	0.8726
U-Net++^11^	0.9593	0.8810	0.8918	0.9739	0.8864	0.8737
MultiResUNet^18^	0.9144	0.6899	**0.9438**	0.9080	0.7971	0.7799
Residual U-Net^59^	0.9367	0.8238	0.8203	0.9620	0.8221	0.8119
TransUNet^22^	0.9626	0.8922	0.8990	0.9764	0.8956	0.8832
UCTransNet^38^	0.9623	0.8991	0.8879	0.9784	0.8935	0.8813
MISSFormer^26^	0.9425	0.8666	0.8007	0.9733	0.8324	0.8229
ParaTransCNN^39^	0.9597	0.8797	0.8964	0.9734	0.8880	0.8753
HiFormer-L^24^	0.9507	0.8133	0.9387	0.9533	0.8715	0.8565
DAEFormer^41^	0.9618	0.9004	0.8830	0.9788	0.8916	0.8795
**DCF-Net (ours)**	**0.9663**	**0.9064**	0.9040	**0.9798**	**0.9052**	**0.8933**

Similarly, on the ISIC 2017 skin lesion segmentation dataset, as shown in Table 3, DCF-Net achieves the best performance on five out of six evaluation metrics. Notably, its Dice score of 90.52% significantly surpasses strong baselines such as TransUNet (89.56%) and DAEFormer (89.16%), reaffirming the robust generalization capability of the proposed hybrid model in medical image segmentation tasks.

### Qualitative results

**Fig. 4:**
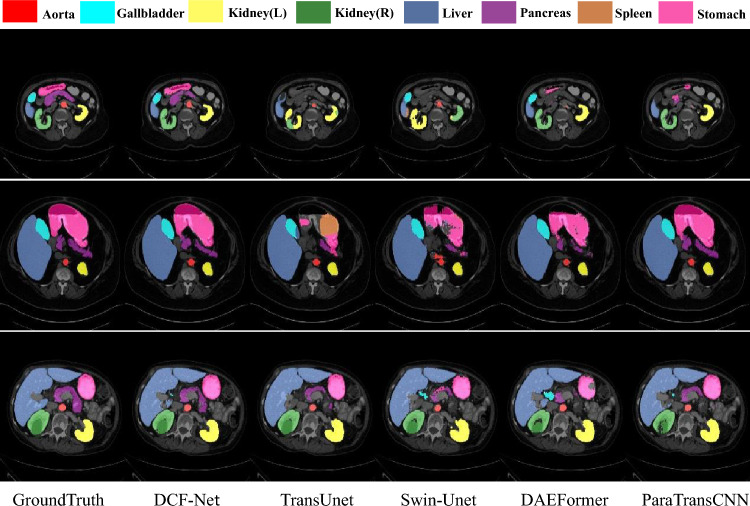
Visual comparison of different methods on the Synapse dataset.

**Fig. 5:**
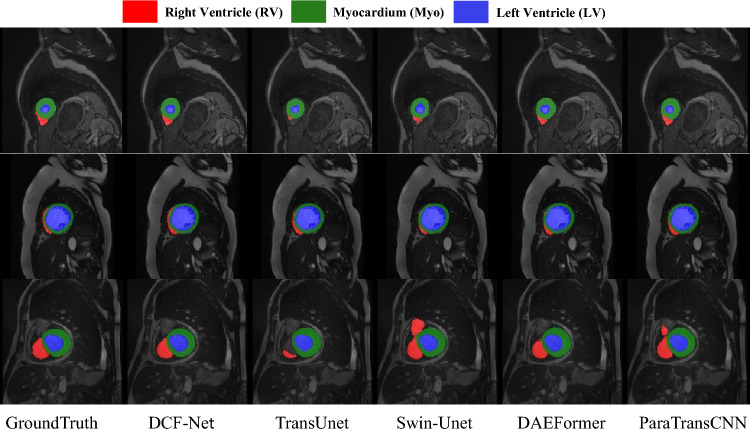
Visual comparison of different methods on the ACDC dataset.

**Fig. 6:**
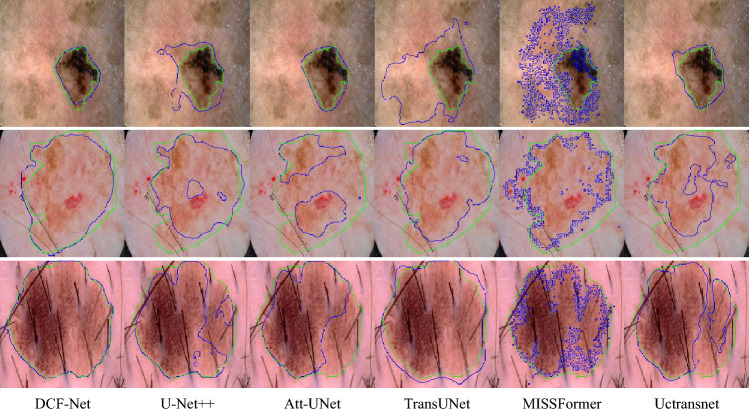
Visual comparison of different methods on the ISIC 2017 dataset, with ground-truth boundaries displayed in green and predicted boundaries in blue.

To further evaluate model performance, we conduct qualitative comparative analyses of segmentation results on the Synapse, ACDC, and ISIC 2017 datasets (Figs. 4-6).

For the Synapse multi-organ segmentation task (Fig. 4), DCF-Net demonstrates superior performance in segmenting anatomically challenging organs (e.g., gallbladder, pancreas, stomach). Its segmentation contours more closely align with true anatomical structures and better preserve boundary completeness. This advantage is driven by the CASA module, which leverages channel-wise XCA to enhance semantic relevance of critical organs and employs TKSA to suppress non-critical region interference, significantly improving boundary continuity for small organs.

In the ACDC cardiac MRI segmentation task, DCF-Net achieves precise delineation of the right ventricle (RV), even in cases where only marginal boundary information is available (as shown in Fig. 5). This is attributed to the SSCF module, which leverages spatially rich shallow features to guide the localization of deep semantic features, while effectively suppressing background interference during ventricular segmentation.

On the ISIC 2017 dermoscopic dataset (Fig. 6), the predicted lesion boundaries (in blue) exhibit high consistency with expert annotations (in green), particularly in cases involving irregular pigmentation and ambiguous borders. Notably, it outperforms pretraining-dependent baselines (e.g., TransUNet) and pure Transformer architectures that require more training epochs to converge (e.g., MISSFormer). This efficiency is attributed to the design of our network, which integrates the local inductive bias of CNNs with the long-range dependency modeling capability of Transformers, enabling superior performance within shorter training cycles.

### Ablation study

Ablation studies on the Synapse dataset (Table 4) were conducted to quantify the individual and combined contributions of the CPCA-Block, CASA module, and SSCF module to overall segmentation performance. The results show that the joint use of CASA and SSCF yields significant improvements, achieving the best results in both Dice score and HD95. Notably, replacing the CPCA-Block with the CASA module to construct a pure Transformer encoder leads to suboptimal performance, confirming that the proposed hybrid design offers superior feature representation and more effective heterogeneous feature fusion than pure Transformer architectures.

To investigate the impact of different encoder resolution settings on model performance, we conducted an ablation study, as shown in Table 5. The results reveal that when the encoder undergoes three downsampling stages, the initial downsampling factor of 1/8 leads to a decline in model performance. This is likely due to the loss of detailed information from the original image caused by the high initial downsampling ratio. On the other hand, when the encoder undergoes four downsampling stages, overall performance also drops. This is attributed to the relatively low input image resolution ($224 \times 224$), where repeated downsampling results in the loss of deep semantic information, which negatively impacts the subsequent feature fusion in the decoder.

**Table 4:** Ablation study on the Synapse dataset, analyzing the impact of key components in DCF-Net. “No” under CPCA-Block denotes the use of a pure Transformer encoder constructed with the CASA dual-attention module, without CNN-based components.

Components				
CPCA-Block	CASA	SSCF	Dice $\uparrow$	HD $\downarrow$
No	Yes	Yes	82.21	17.52
Yes	No	No	82.73	14.49
Yes	Yes	No	83.46	16.12
Yes	No	Yes	83.47	16.00
Yes	Yes	Yes	**84.44**	**14.02**

Significant values are in bold.

**Table 5:** Ablation study on different encoder resolution configurations on the Synapse dataset.

Encoder’s Resolution Config	Dice $\uparrow$	HD $\downarrow$
[1/8, 1/16, 1/32]	82.41	14.16
[1/4, 1/8, 1/16]	**84.44**	**14.02**
[1/4, 1/8, 1/16, 1/32]	83.84	14.96

Significant values are in bold.

**Table 6:** Performance comparison of different CASA-Block stacking configurations in the decoder on the Synapse dataset. The notation [M, N] indicates the number of stacked CASA-Blocks in the two decoder stages, from lower-level (closer to the bottleneck) to higher-level (closer to the output).

Decoder Config	Dice $\uparrow$	HD $\downarrow$
CASA-Block [1, 1]	83.38	16.08
CASA-Block [1, 2]	83.43	17.59
CASA-Block [2, 1]	83.50	15.57
CASA-Block [2, 2]	**84.44**	**14.02**
CASA-Block [2, 3]	83.61	16.64
CASA-Block [3, 2]	83.77	15.51
CASA-Block [3, 3]	84.01	16.38

Significant values are in bold.

To identify the optimal stacking configuration of CASA-Blocks in the decoder, we systematically evaluated various combinations under consistent experimental settings. As shown in Table 6, insufficient stacking fails to fully extract channel-wise semantic semantic features from deep representations, whereas excessive stacking tends to over-filter useful information, thereby weakening the SSCF module’s fusion capability. The results suggest that balanced attention depth across decoder stages is crucial for effective feature interaction. The^2,2^ configuration achieves the best performance, highlighting the benefit of evenly distributing channel attention throughout the decoder.

To further evaluate the design of the SSCF module, we performed a detailed ablation study on three key components: the core attention block, the feature aggregation strategy, and the channel compression method, as shown in Table 7. Replacing the enhanced SCSA-Block with the original SCSA module resulted in a 2.02% decrease in Dice score, confirming the effectiveness of the dual residual design in promoting cross-level feature interaction via skip connections. Additionally, substituting the DWConv compression with a linear projection led to a 1.83% drop, indicating that DWConv is better suited for capturing fused features from encoder and decoder branches. Finally, altering the aggregation strategy from channel-wise concatenation to element-wise addition also impaired performance, suggesting that concatenation better preserves semantic richness from both sides, providing a stronger foundation for cross-semantic fusion in SCSA-Block. These results collectively support the design rationale of the SSCF module.

**Fig. 7:**
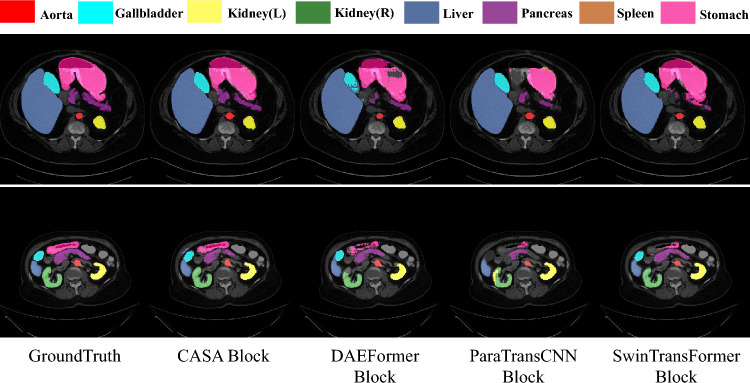
Visual comparison of different decoder’s attention model on the Synapse dataset.

To validate the importance of CASA as a decoder attention module, we conducted an ablation study by replacing CASA with decoder modules from other methods, while preserving the original structure. The results are shown in Table 8. Regardless of the comparison with DAEFormer, which also incorporates a dual attention mechanism, or ParaTransCNN, which employs a hybrid architecture, DCF-Net with CASA consistently achieved the best performance. As illustrated in Fig. 7, our method demonstrates superior segmentation, particularly for critical anatomical regions (e.g., gallbladder, pancreas, stomach), highlighting CASA’s ability to focus on key anatomical areas.

**Table 7:** Ablation study on the Synapse dataset analyzing the key components of the SSCF module. Under Aggregation, “Concatenation” denotes channel-wise fusion of encoder and decoder features, while “Addition” refers to element-wise summation. Main Module indicates the core structure used: “SCSA” refers to the original attention module, and “SCSA-Block” denotes the dual-residual enhanced version. Compression represents the channel reduction method, with “DWConv” and “Linear” indicating depthwise convolution and linear projection, respectively.

Aggregation	Main Module	Compression	Dice $\uparrow$	HD $\downarrow$
Concatenation	SCSA	DWConv	82.42	18.41
Concatenation	SCSA-Block	Linear	82.61	19.03
Addition	SCSA-Block	–	83.76	16.35
Concatenation	SCSA-Block	DWConv	**84.44**	**14.02**

Significant values are in bold.

**Table 8:** Performance comparison of different decoder’s attention model on the Synapse dataset.

Decoder’s Attention Model	Dice $\uparrow$	HD $\downarrow$
+ SwinTransFormer Block	82.42	18.73
+ DAEFormer Block	82.63	15.24
+ ParaTransCNN Block	83.44	23.46
+ CASA Block	**84.44**	**14.02**

Significant values are in bold.

To investigate the impact of TKSA’s dynamic sparsity strategy on model performance, we conducted ablation experiments using different sparsity rates, as shown in Table 9. The results indicate that when the overall sparsity is too high, important semantic information is discarded, leading to a degradation in model performance. Conversely, when the sparsity is too low, irrelevant and unnecessary features are introduced, which also results in a decline in performance.

To verify the effectiveness and structural design of the proposed dual-attention module CASA, we conducted ablation experiments on the Synapse dataset to evaluate the individual contributions of XCA and TKSA, as well as the impact of their combination order, as shown in Table 10. The results indicate that using either XCA or TKSA alone leads to performance degradation, whereas only their combination achieves optimal segmentation results. Among the variants, the sequential configuration with XCA followed by TKSA (“Channel-pre”) outperforms both the parallel arrangement and the reversed order (“Sparse-pre”), demonstrating that initial feature filtering via channel attention followed by sparse refinement yields the most effective attention interaction design for medical image segmentation.

**Table 9:** Ablation study on different sparsity rate of TKSA on the Synapse dataset.

TKSA’s Sparsity Rate	Dice $\uparrow$	HD $\downarrow$
[1/4, 1/3, 1/2, 2/3]	82.86	18.37
[1/3, 1/2, 2/3, 3/4]	83.15	18.42
[1/2, 2/3, 3/4, 4/5]	**84.44**	**14.02**
[2/3, 3/4, 5/6, 7/8]	83.59	15.12

Significant values are in bold.

**Table 10:** Ablation study of CASA on the Synapse dataset, where “Parallel” denotes parallel placement of XCA and TKSA. “Channel-only” and “Sparse-only” represent the individual use of XCA and TKSA, respectively. “Sparse-pre” refers to the sequential configuration with TKSA preceding XCA, while “Channel-pre” indicates the reverse.

Method	Dice $\uparrow$	HD $\downarrow$
Parallel	83.21	16.78
Channel-only	83.06	18.13
Sparse-only	83.47	16.96
Sparse-pre	83.80	14.83
Channel-pre	**84.44**	**14.02**

Significant values are in bold.

## Limitations and future work

While DCF-Net demonstrates promising results in medical image segmentation, several limitations remain that need to be addressed.

First, although we have validated the effectiveness of our model on the Synapse, ACDC, and ISIC2017 datasets, further evaluation on larger-scale and more diverse datasets is essential to better assess the model’s generalization capabilities. Future experiments should involve datasets from a broader range of medical imaging tasks, such as cell segmentation and brain tumor datasets. Moreover, due to computational constraints, this work has primarily focused on 2D slice-based segmentation, leaving the potential for volumetric 3D medical imaging scenarios (e.g., brain MRI or lung CT) underexplored.

Second, although DCF-Net demonstrates strong segmentation performance compared to many pure Transformer or hybrid models, its model size, computational cost, and efficiency do not yet provide significant improvements over existing methods. Consequently, the model may still encounter latency bottlenecks in real-time clinical applications, particularly those requiring rapid inference, such as intraoperative navigation.

Third, the model’s performance remains sensitive to the availability of annotated data. In resource-constrained environments, such as rare disease detection, the lack of abundant annotated datasets may limit the generalization of the model. Semi-supervised or few-shot learning approaches could offer a viable solution to improve performance under such conditions.

To address the aforementioned limitations, our future work will focus on the following directions:

- Lightweight Architectures: We plan to explore more lightweight architectures to further reduce computational complexity and accelerate inference, aiming for real-time deployment in clinical settings.
- Semi-Supervised Learning: We will investigate the integration of semi-supervised learning methods to adapt our model to scenarios with limited labeled data, making it more robust in environments where annotated data is scarce.
- Expansion to 3D and More Diverse Datasets: We aim to extend DCF-Net to 3D medical volume segmentation, leveraging advanced hardware to enable the accurate modeling of spatial continuity across slices. Additionally, we will evaluate the model on a broader set of medical image datasets to test its applicability in diverse clinical domains.
- Optimization Strategies: We will investigate the impact of different hyperparameter optimization strategies on the performance across various datasets and explore dynamic optimization approaches, including gradient-based neurodynamic optimization^60,61^ and coevolutionary mechanisms^62–64^ for nonlinear optimization applications.
- Cross-Domain Technical Synergy: The edge blurring phenomenon encountered in super-resolution tasks^65,66^ demonstrates significant commonality with the weak boundary challenges prevalent in medical image segmentation. We intend to investigate the incorporation of super-resolution-derived methodologies to enhance medical image segmentation.

## Conclusions

In this paper, we propose DCF-Net, a dual-attention cross-layer fusion network that combines the complementary strengths of CNNs and Transformers. Specifically, we introduce two core modules, CASA and SSCF, to address the challenges of limited representation of fine-grained anatomical details and inefficient fusion of heterogeneous features in medical image segmentation. Extensive experiments demonstrate that DCF-Net consistently outperforms superior methods on both radiological and dermatological benchmarks. Visualization results further show that our method excels in segmenting anatomically challenging regions–especially those with irregular boundaries, low contrast, or limited boundary cues–offering superior boundary precision and overall segmentation quality.

## Data Availability

All datasets used in this study are publicly available. The Synapse Multi-Organ Segmentation Dataset is accessible at https://www.synapse.org/Synapse:syn3193805/wiki/217789, the Automated Cardiac Diagnostic Challenge Dataset can be found at https://www.creatis.insa-lyon.fr/Challenge/acdc/, and the International Skin Imaging Collaboration Dataset is available at https://challenge.isic-archive.com/data/#2017.

## References

[1] Gao, J., Jiang, Q., Zhou, B. & Chen, D. Convolutional neural networks for computer-aided detection or diagnosis in medical image analysis: An overview. *Math. Biosci. Eng.* **16**, 6536–6561 (2019). 31698575 10.3934/mbe.2019326

[2] Antonelli, M. et al. The medical segmentation decathlon. *Nat. Commun.* **13**, 4128 (2022). 35840566 10.1038/s41467-022-30695-9 PMC9287542

[3] Litjens, G. et al. A survey on deep learning in medical image analysis. *Med. Image Anal.* **42**, 60–88 (2017). 28778026 10.1016/j.media.2017.07.005

[4] Lou, M. & Yu, Y. Overlock: An overview-first-look-closely-next convnet with context-mixing dynamic kernels. In *Proceedings of the Computer Vision and Pattern Recognition Conference*, 128–138 (2025).

[5] Fu, Y., Lou, M. & Yu, Y. Segman: Omni-scale context modeling with state space models and local attention for semantic segmentation. In *Proceedings of the Computer Vision and Pattern Recognition Conference*, 19077–19087 (2025).

[6] Qureshi, I. et al. Medical image segmentation using deep semantic-based methods: A review of techniques, applications and emerging trends. *Inf. Fusion* **90**, 316–352 (2023).

[7] Xiao, H., Li, L., Liu, Q., Zhu, X. & Zhang, Q. Transformers in medical image segmentation: A review. *Biomed. Signal Process. Control* **84**, 104791 (2023).

[8] Zhang, Y., Shen, Z. & Jiao, R. Segment anything model for medical image segmentation: Current applications and future directions. *Comput. Biol. Medicine* **171**, 108238 (2024). 10.1016/j.compbiomed.2024.108238 38422961

[9] Azad, R. et al. Medical image segmentation review: The success of u-net. *IEEE Trans. Pattern Anal. Mach. Intell.* **46**(12), 10076-10095 (2024). 10.1109/TPAMI.2024.3435571 39167505

[10] Ronneberger, O., Fischer, P. & Brox, T. U-net: Convolutional networks for biomedical image segmentation. In *Medical image computing and computer-assisted intervention–MICCAI 2015: 18th international conference, Munich, Germany, October 5-9, 2015, proceedings, part III 18*, 234–241 (Springer, 2015).

[11] Zhou, Z., Rahman Siddiquee, M. M., Tajbakhsh, N. & Liang, J. Unet++: A nested u-net architecture for medical image segmentation. In *Deep learning in medical image analysis and multimodal learning for clinical decision support: 4th international workshop, DLMIA 2018, and 8th international workshop, ML-CDS 2018, held in conjunction with MICCAI 2018, Granada, Spain, September 20, 2018, proceedings 4*, 3–11 (Springer, 2018). 10.1007/978-3-030-00889-5_1 PMC7329239 32613207

[12] Huang, H. et al. Unet 3+: A full-scale connected unet for medical image segmentation. In *ICASSP 2020-2020 IEEE international conference on acoustics, speech and signal processing (ICASSP)*, 1055–1059 (IEEE, 2020). 10.1109/ICASSP40776.2020.9053555 PMC7543994 33041676

[13] Jin, Q. et al. Dunet: A deformable network for retinal vessel segmentation. *Knowl.-Based Syst.* **178**, 149–162 (2019).

[14] Li, X. et al. H-denseunet: hybrid densely connected unet for liver and tumor segmentation from ct volumes. *IEEE Trans. Med. Imaging* **37**, 2663–2674 (2018). 29994201 10.1109/TMI.2018.2845918

[15] Fan, D.-P. et al. Pranet: Parallel reverse attention network for polyp segmentation. In *International conference on medical image computing and computer-assisted intervention*, 263–273 (Springer, 2020).

[16] Jin, Q., Meng, Z., Sun, C., Cui, H. & Su, R. Ra-unet: A hybrid deep attention-aware network to extract liver and tumor in ct scans. *Front. Bioeng. Biotechnol.* **8**, 605132 (2020). 33425871 10.3389/fbioe.2020.605132 PMC7785874

[17] Oktay, O. et al. Attention u-net: Learning where to look for the pancreas. *arXiv preprint* arXiv:1804.03999 (2018).

[18] Ibtehaz, N. & Rahman, M. S. Multiresunet: Rethinking the u-net architecture for multimodal biomedical image segmentation. *Neural Netw.* **121**, 74–87 (2020). 31536901 10.1016/j.neunet.2019.08.025

[19] Rahman, M. M., Munir, M. & Marculescu, R. Emcad: Efficient multi-scale convolutional attention decoding for medical image segmentation. In *Proceedings of the IEEE/CVF Conference on Computer Vision and Pattern Recognition*, 11769–11779 (2024).

[20] Vaswani, A. et al. Attention is all you need. *Adv. Neural Inf. Process. Syst.* **30** (2017).

[21] Dosovitskiy, A. et al. An image is worth 16x16 words: Transformers for image recognition at scale. *arXiv preprint* arXiv:2010.11929 (2020).

[22] Chen, J. et al. Transunet: Transformers make strong encoders for medical image segmentation. *arXiv preprint* arXiv:2102.04306 (2021).

[23] Xie, Y., Zhang, J., Shen, C. & Xia, Y. Cotr: Efficiently bridging cnn and transformer for 3d medical image segmentation. In *Medical Image Computing and Computer Assisted Intervention–MICCAI 2021: 24th International Conference, Strasbourg, France, September 27–October 1, 2021, Proceedings, Part III 24*, 171–180 (Springer, 2021).

[24] Heidari, M. et al. Hiformer: Hierarchical multi-scale representations using transformers for medical image segmentation. In *Proceedings of the IEEE/CVF winter conference on applications of computer vision*, 6202–6212 (2023).

[25] Rahman, M. M. & Marculescu, R. Multi-scale hierarchical vision transformer with cascaded attention decoding for medical image segmentation. In *Medical Imaging with Deep Learning*, 1526–1544 (PMLR, 2024).

[26] Huang, X., Deng, Z., Li, D., Yuan, X. & Fu, Y. Missformer: An effective transformer for 2d medical image segmentation. *IEEE Trans. Med. Imaging* **42**, 1484–1494 (2022). 10.1109/TMI.2022.3230943 37015444

[27] Azad, R. et al. Transdeeplab: Convolution-free transformer-based deeplab v3+ for medical image segmentation. In *International Workshop on PRedictive Intelligence In MEdicine*, 91–102 (Springer, 2022).

[28] Landman, B. et al. Multi-atlas labeling beyond the cranial vault, 10.7303/syn3193805 (2015). **Dataset ID: syn3193805**.

[29] Bernard, O. et al. Deep learning techniques for automatic mri cardiac multi-structures segmentation and diagnosis: is the problem solved?. *IEEE Trans. Med. Imaging* **37**, 2514–2525 (2018). 29994302 10.1109/TMI.2018.2837502

[30] Codella, N. C. et al. Skin lesion analysis toward melanoma detection: A challenge at the 2017 international symposium on biomedical imaging (isbi), hosted by the international skin imaging collaboration (isic). In *2018 IEEE 15th international symposium on biomedical imaging (ISBI 2018)*, 168–172 (IEEE, 2018).

[31] Cao, H. et al. Swin-unet: Unet-like pure transformer for medical image segmentation. In *European conference on computer vision*, 205–218 (Springer, 2022).

[32] Liu, Z. et al. Swin transformer: Hierarchical vision transformer using shifted windows. In *Proceedings of the IEEE/CVF international conference on computer vision*, 10012–10022 (2021).

[33] Lou, M. et al. Transxnet: learning both global and local dynamics with a dual dynamic token mixer for visual recognition. *IEEE Trans. Neural Netw. Learn. Syst.* **36**(6), 11534-11547 (2025). 10.1109/TNNLS.2025.3550979 40178959

[34] Bao, L. et al. Aggregating transformers and cnns for salient object detection in optical remote sensing images. *Neurocomputing* **553**, 126560 (2023).

[35] Bao, L. et al. Ifenet: Interaction, fusion, and enhancement network for vdt salient object detection. *IEEE Trans. Image Process.* **34**, 483-494 (2025). 10.1109/TIP.2025.3527372 40031013

[36] Huang, H. et al. Channel prior convolutional attention for medical image segmentation. *Comput. Biol. Med.* **178**, 108784 (2024). 38941900 10.1016/j.compbiomed.2024.108784

[37] Zhang, Z., Xu, C., Li, Z., Chen, Y. & Nie, C. Multi-scale fusion semantic enhancement network for medical image segmentation. *Sci. Rep.* **15**, 23018 (2025). 40594784 10.1038/s41598-025-07806-9 PMC12214572

[38] Wang, H., Cao, P., Wang, J. & Zaiane, O. R. Uctransnet: rethinking the skip connections in u-net from a channel-wise perspective with transformer. *Proc. AAAI Conf. Artif. Intell.* **36**, 2441–2449 (2022).

[39] Sun, H., Xu, J. & Duan, Y. Paratranscnn: Parallelized transcnn encoder for medical image segmentation. *arXiv preprint* arXiv:2401.15307 (2024).

[40] Bougourzi, F., Dornaika, F., Taleb-Ahmed, A. & Truong Hoang, V. Rethinking attention gated with hybrid dual pyramid transformer-cnn for generalized segmentation in medical imaging. In *International Conference on Pattern Recognition*, 243–258 (Springer, 2024).

[41] Azad, R., Arimond, R., Aghdam, E. K., Kazerouni, A. & Merhof, D. Dae-former: Dual attention-guided efficient transformer for medical image segmentation. In *International workshop on predictive intelligence in medicine*, 83–95 (Springer, 2023).

[42] Yang, W. et al. A dual encoder network with multiscale feature fusion and multiple pooling channel spatial attention for skin scar image segmentation. *Sci. Rep.* **15**, 22810 (2025). 40594041 10.1038/s41598-025-05239-y PMC12217583

[43] Dai, Y., Gieseke, F., Oehmcke, S., Wu, Y. & Barnard, K. Attentional feature fusion. In *Proceedings of the IEEE/CVF winter conference on applications of computer vision*, 3560–3569 (2021).

[44] Zhang, S., Xu, Y., Wu, Z. & Wei, Z. Ctc-net: A novel coupled feature-enhanced transformer and inverted convolution network for medical image segmentation. In *Asian Conference on Pattern Recognition*, 273–283 (Springer, 2023).

[45] Chen, Y., Lu, X. & Xie, Q. Atformer: Advanced transformer for medical image segmentation. *Biomed. Signal Process. Control* **85**, 105079 (2023).

[46] Lou, M., Fu, Y. & Yu, Y. Sparx: A sparse cross-layer connection mechanism for hierarchical vision mamba and transformer networks. *Proc. AAAI Conf. Artif. Intell.* **39**, 19104–19114 (2025).

[47] Chen, X., Li, H., Li, M. & Pan, J. Learning a sparse transformer network for effective image deraining. In *Proceedings of the IEEE/CVF conference on computer vision and pattern recognition*, 5896–5905 (2023).

[48] Ali, A. et al. Xcit: Cross-covariance image transformers. *Adv. Neural Inf. Process. Syst.* **34**, 20014–20027 (2021).

[49] Xie, E. et al. Segformer: Simple and efficient design for semantic segmentation with transformers. *Adv. Neural Inf. Process. Syst.* **34**, 12077–12090 (2021).

[50] Si, Y. et al. Scsa: Exploring the synergistic effects between spatial and channel attention. *Neurocomputing* **634**, 129866 (2025).

[51] Zhu, W. et al. Selfreg-unet: Self-regularized unet for medical image segmentation. In *International Conference on Medical Image Computing and Computer-Assisted Intervention*, 601–611 (Springer, 2024). 10.1007/978-3-031-72111-3_56 PMC12408486 40917447

[52] Rahman, M. M. & Marculescu, R. Medical image segmentation via cascaded attention decoding. In *Proceedings of the IEEE/CVF winter conference on applications of computer vision*, 6222–6231 (2023).

[53] Ruan, J., Li, J. & Xiang, S. Vm-unet: Vision mamba unet for medical image segmentation. *arXiv preprint* arXiv:2402.02491 (2024).

[54] Rahman, M. M. & Marculescu, R. G-cascade: Efficient cascaded graph convolutional decoding for 2d medical image segmentation. In *Proceedings of the IEEE/CVF Winter Conference on Applications of Computer Vision*, 7728–7737 (2024).

[55] Hatamizadeh, A. et al. Unetr: Transformers for 3d medical image segmentation. In *Proceedings of the IEEE/CVF winter conference on applications of computer vision*, 574–584 (2022).

[56] Xu, G., Zhang, X., He, X. & Wu, X. Levit-unet: Make faster encoders with transformer for medical image segmentation. In *Chinese Conference on Pattern Recognition and Computer Vision (PRCV)*, 42–53 (Springer, 2023).

[57] Wang, H. et al. Mixed transformer u-net for medical image segmentation. In *ICASSP 2022-2022 IEEE international conference on acoustics, speech and signal processing (ICASSP)*, 2390–2394 (IEEE, 2022).

[58] Perera, S., Navard, P. & Yilmaz, A. Segformer3d: an efficient transformer for 3d medical image segmentation. In *Proceedings of the IEEE/CVF Conference on Computer Vision and Pattern Recognition*, 4981–4988 (2024).

[59] Zhang, Z., Liu, Q. & Wang, Y. Road extraction by deep residual u-net. *IEEE Geosci. Remote Sens. Lett.* **15**, 749–753 (2018).

[60] Fan, J. et al. Coevolutionary neural dynamics considering multiple strategies for nonconvex optimization. *Tsinghua Sci. Technol.*10.26599/TST.2025.9010120(2025).

[61] Liu, M., Chen, L., Du, X., Jin, L. & Shang, M. Activated gradients for deep neural networks. *IEEE Trans. Neural Netw. Learn. Syst.* **34**, 2156–2168 (2021). 10.1109/TNNLS.2021.3106044 34469312

[62] Liu, M., Li, Y., Chen, Y., Qi, Y. & Jin, L. A distributed competitive and collaborative coordination for multirobot systems. *IEEE Trans. Mob. Comput.* **23**, 11436–11448 (2024).

[63] Huang, H., Jin, L. & Zeng, Z. A momentum recurrent neural network for sparse motion planning of redundant manipulators with majorization-minimization. *IEEE Trans. Ind. Electron.*10.1109/TIE.2025.3566731 (2025).

[64] Jin, L., Wei, L. & Li, S. Gradient-based differential neural-solution to time-dependent nonlinear optimization. *IEEE Trans. Autom. Control* **68**, 620–627 (2022).

[65] Talreja, J., Aramvith, S. & Onoye, T. Dans: Deep attention network for single image super-resolution. *IEEE Access* **11**, 84379–84397 (2023).

[66] Talreja, J., Aramvith, S. & Onoye, T. Dhtcun: deep hybrid transformer cnn u network for single-image super-resolution. *IEEE Access* **12**, 122624-122641 (2024).

